# Proteome profiling reveals insights into cold-tolerant growth in sea buckthorn

**DOI:** 10.1186/s12953-016-0103-z

**Published:** 2016-10-07

**Authors:** Caiyun He, Guori Gao, Jianguo Zhang, Aiguo Duan, Hongmei Luo

**Affiliations:** 1 State Key Laboratory of Tree Genetics and Breeding, Key Laboratory of Tree Breeding and Cultivation of the State Forestry Administration, Research Institute of Forestry, Chinese Academy of Forestry, Beijing, People’s Republic of China; 2 Collaborative Innovation Center of Sustainable Forestry in Southern China, Nanjing Forestry University, Nanjing, People’s Republic of China; 3 Experimental Center of Desert Forestry, Chinese Academy of Forestry, InnerMonglia, People’s Republic of China

**Keywords:** Antioxidative systems, DIGE, *Hippophae rhamnoides*, Low temperature, Photosynthesis, Post-translational modification

## Abstract

**Background:**

Low temperature is one of the crucial environmental factors limiting the productivity and distribution of plants. Sea buckthorn (*Hippophae rhamnoides* L.), a well recognized multipurpose plant species, live successfully in in cold desert regions. But their molecular mechanisms underlying cold tolerance are not well understood.

**Methods:**

Physiological and biochemical responses to low-temperature stress were studied in seedlings of sea buckthorn. Differentially expressed protein spots were analyzed using multiplexing fluorescent two-dimensional fluorescence difference gel electrophoresis (2D-DIGE) coupled with matrix-assisted laser desorption/ionization (MALDI) time-of-flight/time-of-flight (TOF/TOF) mass spectrometry (MS), the concentration of phytohormone was measured using enzyme-linked immunosorbent assay, and a spectrophotometric assay was used to measure enzymatic reactions.

**Results:**

With the increase of cold stress intensity, the photosynthesis rate, transpiration rate, stomatal conductance in leaves and contents of abscisic acid (ABA) and indole acetic acid (IAA) in roots decreased significantly; however, water-use efficiency, ABA and zeatin riboside in leaves increased significantly, while cell membrane permeability, malondialdehyde and IAA in leaves increased at 7 d and then decreased at 14 d. DIGE and MS/MS analysis identified 32 of 39 differentially expressed protein spots under low-temperature stress, and their functions were mainly involved in metabolism, photosynthesis, signal transduction, antioxidative systems and post-translational modification.

**Conclusion:**

The changed protein abundance and corresponding physiological–biochemical response shed light on the molecular mechanisms related to cold tolerance in cold-tolerant plants and provide key candidate proteins for genetic improvement of plants.

**Electronic supplementary material:**

The online version of this article (doi:10.1186/s12953-016-0103-z) contains supplementary material, which is available to authorized users.

## Background

Plants being sessile have developed intricate adaptation mechanisms to stresses. Among abiotic environmental stresses, low temperature is one of the crucial factors limiting plant productivity and distribution [1–3]. Therefore, identifying the reasons behind cold adaptation in cold-tolerant plants could be of central importance to gaining a deeper understanding of the mechanisms involved, and be beneficial for plant growth and production [4, 5].

Sea buckthorn (*Hippophae rhamnoides*) is well adapted to extreme temperatures ranging from −43 to 40 °C [6], and it is distributed in cold regions of Russia, China, Finland, Sweden and many other countries of Asia, Europe and North America [7], which provided an ideal material to study mechanisms related to low-temperature (LT) stress tolerance [2]. Although there is a considerable wealth of studies on origin, distribution, ecology and nutrient and medicinal values in *Hippophae* [8–15], the physiological and biochemical basis of tolerance and mechanisms of abiotic stress response, especially low-temperature or cold response, are not well understood [2, 16, 17].

Due to the direct roles of proteins in plant stress responses, profound changes in proteome composition can be observed during plant acclimation to stress. Mass spectrometry (MS)-based proteomics has become an essential tool in unraveling possible relationships between protein abundance and plant stress acclimation [18, 19]. The present study discusses the proteome-wide protein responses to low-temperature stress of *H. rhamnoides* cv. ‘Chuisk’, a widely cultivated hybrid of *H. rhamnoides* subsp*. mongolica* and *H. rhamnoides* subsp*. rhamnoides* in northeast China, which has excellent cold resistance and good characteristics, including large berries, high content of oil and high production. Using physiological, biochemical and comparative proteomic analyses, we hope to provide insights into cold adaptation mechanisms in this cold-tolerant species.

## Results

### Physiological and biochemical responses during LT stress

**Fig. 1:**
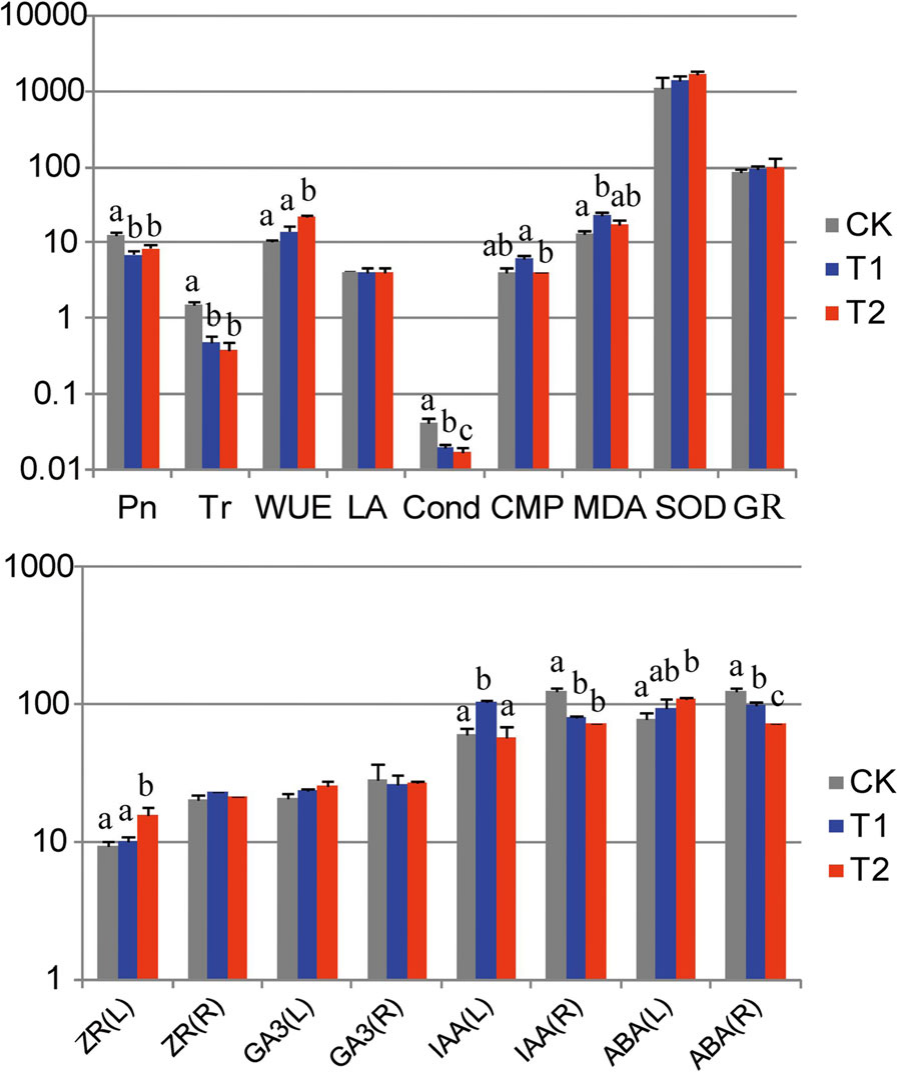
The effect of low-temperature stress on physiological and biochemical characteristics for *H. rhamnoides* cv. ‘Chuisk’ leaves (L) and roots (R): Net photosynthetic rates (Pn), transpiration rate (Tr), stomatal conductance (Cond), superoxide dismutase (SOD), glutathione reductase (GR), malondialdehyde (MDA), Fresh leaf area (LA), cell membrane permeability (CMP), water-use efficiency (WUE), abscisic acid (ABA), indole acetic acid (IAA), gibberellic acid (GA_3_) and zeatin riboside (ZR). CK represents control, T1 and T2 respectively represent 4 °C low temperature treatment for 7 and 14 days. Each histogram represents the mean of three biological replicates. Vertical bars represent SD of the mean (*n* = 3). Different letters on a column with the same pattern indicate significant differences at *P* ≤ 0.05 according to the *t*-test

Under the LT treatments 7 d (T1) and 14 d (T2), the values of superoxide dismutase (SOD), glutathione reductase (GR) and zeatin riboside in root [ZR(R)] showed no significant changes, but leaf area (LA) and gibberellins (GA_3_) did. The decreases in stomatal conductance (Cond) and abscisic acid in root [ABA(R)] were significant with the extension of LT treatment time (*p*-value ≤ 0.05). There were significant increases in water-use efficiency (WUE), ZR (L) (L indicated leaf) and ABA (L) for T1. Gradual decreases in photosynthesis rate (Pn), transpiration rate (Tr) and indole acetic acid [IAA(R)] were observed among both controls and treatments. Cell membrane permeability (CMP), malondialdehyde (MDA) and IAA(L) showed non-consistent changes (increase at T1 and decrease at T2) under LT stress (Fig. 1).

### Variations in LT responsive proteins

**Fig. 2:**
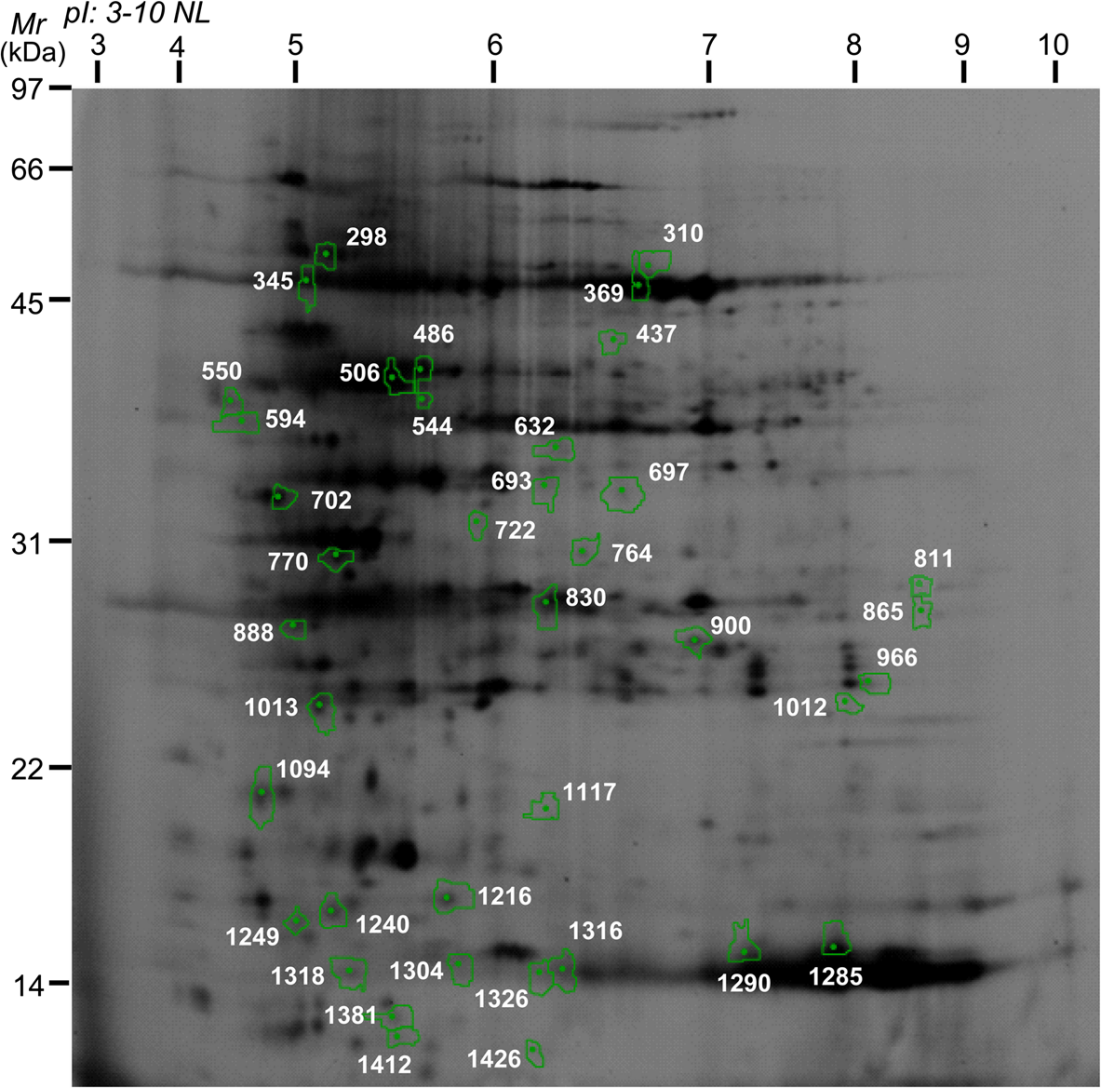
Representative fluorescent dyes-stained two-dimensional gel of leaf proteins in response to low-temperature stress in *H. rhamnoides* cv. ‘Chuisk’ seedlings. The relative *M*_r_ (on the left) and the p*I* (on the top) are given. The white numbers represent 39 differentially expressed protein spots

The DeCyder image analysis of fluorescent images detected 1466 ± 35 protein spots (Fig. 2, Additional file 1: Figure S1). One-way analysis of variance (ANOVA) showed that 39 different protein spots were significantly affected by LT stress (*p*-value ≤ 0.05) (Fig. 2), of which 32 proteins were identified using the MS/MS method: eight exhibited a gradual increase (A-type), five exhibited a gradual decrease (B-type), 11 exhibited an increase at T1 and decrease at T2 (C-type) and eight exhibited a decrease at T1 and increase at T2 (D-type) (Additional file 2: Table S1).

### Gene ontology (GO) and pathway enrichment analysis of differentially expressed protein spots

**Fig. 3:**
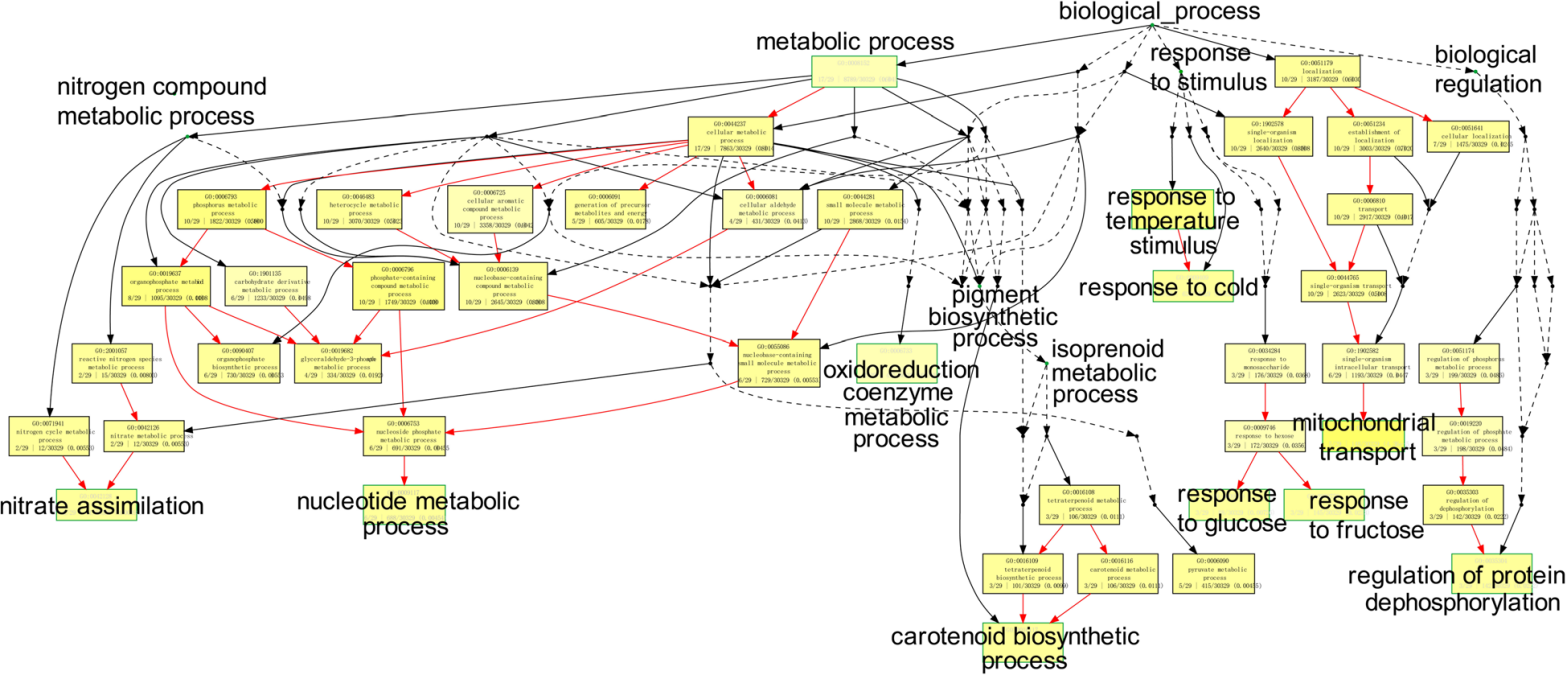
Biological process enrichment clusters for 37 low-temperature stress proteins with *Arabidopsis thaliana* homologues using GOEAST

Ninety-seven GO enrichment terms were obtained using GOEAST [20]. The enriched biological process ontology included the metabolic process (nitrate assimilation and nucleotide metabolic, oxidoreduction coenzyme metabolic and carotenoid biosynthetic processes), biological regulation (regulation of protein dephosphorylation), response to stimulus (responses to cold, glucose and fructose) and localization (mitochondrial transport) (Fig. 3 and Additional file 2: Table S2).

KEGG pathway analysis [21] showed that 18 terms including genetic information processing (folding, sorting and degradation, and translation) and metabolism (amino acid, carbohydrate and energy metabolisms) were enriched under LT stress (Additional file 2: Table S3). Of the 32 identified protein spots, 14 (43.75 %) proteins were classified to metabolism pathways including energy metabolism (14 protein spots), carbohydrate metabolism (12 spots) and amino acid metabolism (spots 486 and 693, C-type; and spot 437, D-type). Energy metabolism was enriched in carbon fixation in photosynthetic organisms including ribulose-bisphosphate carboxylase large chain (rbcL; 10 homologous/isoform protein spots), photosynthesis involving oxygen-evolving enhancer protein 1 (spot 770, A-type), photosystem I reaction center subunit II (spot 966, C-type) and nitrogen metabolism (spot 486, C-type, glutamine synthetase, glnA). Carbohydrate metabolism was enriched in glyoxylate and dicarboxylate metabolism (11 protein spots), and citrate cycle and pyruvate metabolism (spot 693, C-type, Nodule-enhanced malate dehydrogenase). Four of 32 proteins belonged to the pathway of genetic information processing (spots 298 and 345, A-type, TCP-1/cpn60 chaperonin family protein; spot 1117, D-type, 18.5-kDa class I heat shock protein; and spot 722, D-type, unnamed protein) (Additional file 2: Tables S1 and S3).

### mRNA expression of differentially expressed protein spots between different treatments

**Fig. 4:**
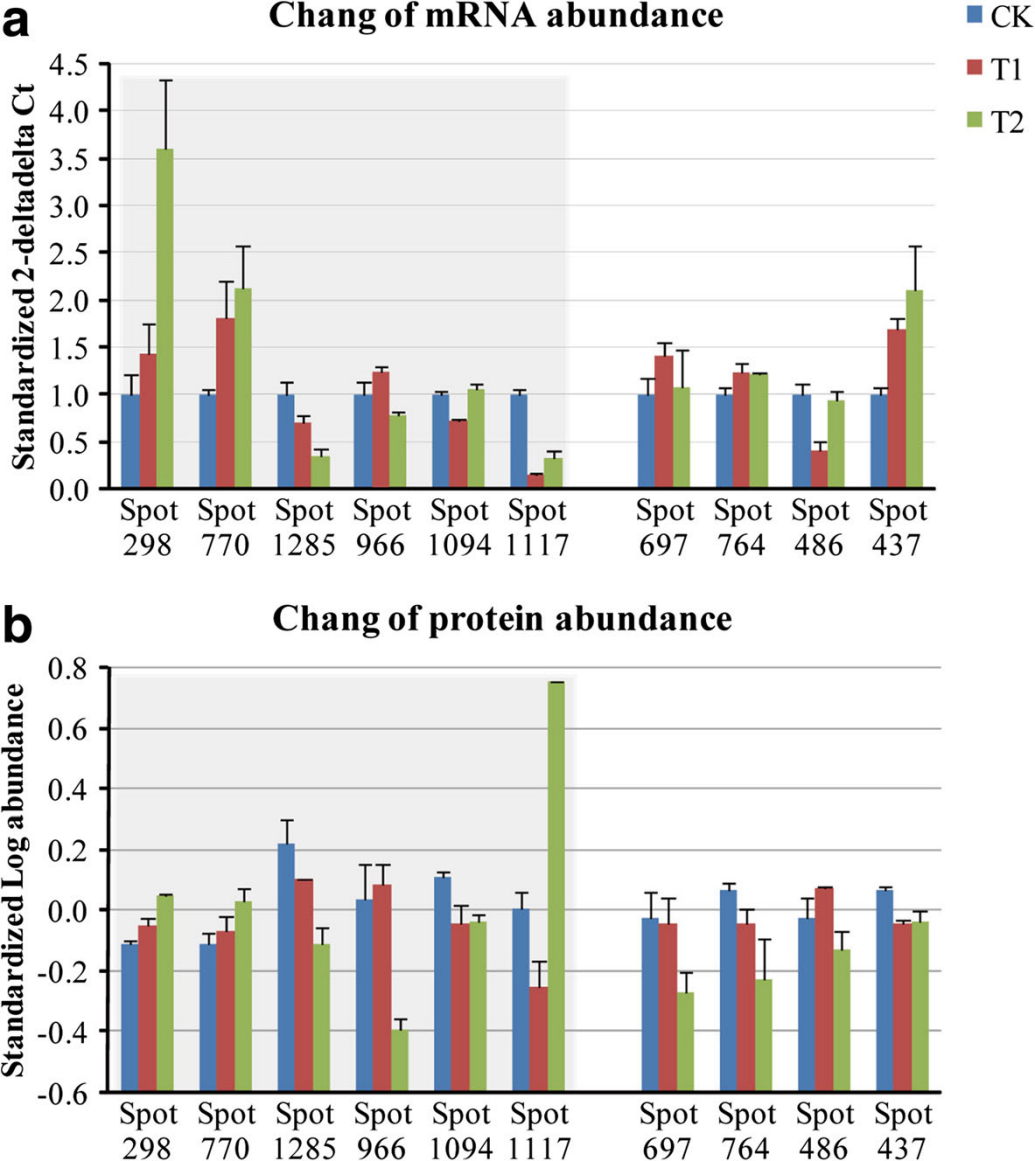
Quantitative RT-PCR of mRNA changes. **a** and **b** showed expression abundance of mRNA and protein we detected between RT-PCR and 2D-DIGE, respectively. Gray margins showed the agreement in the six genes we detected between RT-PCR and 2D-DIGE

To confirm the reliability of the MALDI-TOF MS/MS and 2D-DIGE in detecting *H. rhamnoide*s, we used RealTime PCR to compare the expression levels of identified protein genes. Ten genes were randomly selected from the differentially expressed protein spots in the three groups. Similar expression patterns between protein and mRNA were observed in six of the ten detected genes (Fig. 4). Of which, two were up-regulated (Spots 298 and 770), one was down-regulated (Spot 1285), one was up-regulated under D1 stress and down-regulated under D2 stress (Spot 966), and two was down-regulated under D1 stress and up-regulated under D2 stress (Spots 1094 and 1117).

## Discussion

### Endogenous hormones and antioxidant enzymes play roles in physiological and biochemical responses of sea buckthorn under LT stress

Compared with GA_3_ and ZR, ABA and IAA responded more actively. The LT stress induced the decline of ABA content in sea buckthorn roots, and then accumulation in leaves, which led to stomatal closure and restrained aboveground Pn, Tr and Cond. Meanwhile, the complementary/equilibrium of growth-promoting hormones such as ZR and IAA possibly contributed to maintaining plant growth, while the increase of ABA in leaves also showed growth inhibition under LT stress [22]. The changed protein abundance of probable LRR receptor-like serine/threonine-protein kinase RKF3-like (spot 1426, C-type) involved in hormone-mediated (ABA and GA) signaling pathways, and hypothetical protein (spot 830, C-type) participating in IAA biosynthetic/metabolic process, further showed that ABA, GA and IAA played an important role in responding to LT stress in sea buckthorn, as found in many other cold-stress studies [23–27]. These confirmed that many hormonal proteins contributed to the changes in ABA, GA and IAA content under LT stress and so induced the hormone signaling pathways responding to LT stress in sea buckthorn.

In addition, the CMP and MDA increase under LT stress suggested that they induced cell damage and membrane lipid peroxidation. Some protein spots of antioxidant enzymes, such as zinc-binding dehydrogenase family oxidoreductase (spot 544, A-type), peroxiredoxin family protein (Prx, spot 888, D-type), dehydroascorbate reductase (DHAR, spot 900, C-type) and glnA (spot 486, C-type), changed dynamically in protein abundance under LT stress, suggesting that they had taken part in changes of enzymatic activity or antioxidants (i.e. SOD and GR). Many proteins involved in antioxidative systems were also induced by LT stress in *H. rhamnoides* [2] and *Arabidopsis* [28]. This study hint at cooperation between hormone and redox signaling pathways in responding to LT stress, and this confers enormous regulatory potential because it allows plants to adapt to changing and often challenging stress conditions [29].

### Proteins related to metabolism, signal transduction and post-translational modification contribute to LT stress responses in sea buckthorn

In forest tree species, many genes taking part in metabolic processes were differentially expressed under most abiotic stress conditions [30–34]. Pn significantly decreased under LT stress, and expression of the rbcLs and oxygen-evolving enhancer proteins in sea buckthorn greatly changed consistent with a number of previous reports [35–37]. This suggested negative effects on photosynthetic efficiency and photosynthesis rate. Many studies have shown that LT triggers a cellular signal transduction pathway leading to molecular and metabolic changes [26, 38–40]. This study found glutamine synthetase (spot 486, C-type) was differently expressed during LT stress, suggesting that cold stress stimuli initiated the signal transmission processes in *H. rhamnoide*s. During cold acclimation, plants accumulate specific types of solutes, such as soluble sugars (i.e. glucose and fructose) [41] and carotenoids [42, 43] to enhance freezing tolerance. The different expression of Prx (spot 888, D-type) and glnA (spot 486, C-type) under LT stress suggested the accumulation of soluble sugars in *H. rhamnoide*s. Meanwhile, two proteins (spots 544 and 594, A-type) involving carotenoid biosynthetic process were enriched in the up-regulated differently expressed protein spots, suggesting an important role of carotenoid in responding to LT stress in sea buckthorn.

It is noteworthy that two identified isoforms had multiple spots located at different positions on the gel with different molecular weight, *pI* or both. For example, spots 298 and 345 were both identified as TCP-1/cpn60 chaperonin family protein and spots 369, 550, 764, 811, 865, 1012, 1285, 1290, 1316 and 1326 were rbcL homologs. These isoforms might represent post-translationally modified forms of the same protein, such as phosphorylated and glycosylated forms under LT stress, hinting at the post-translational regulation of cold acclimation responsein *H. rhamnoide*s [44, 45].

**Fig. 5:**
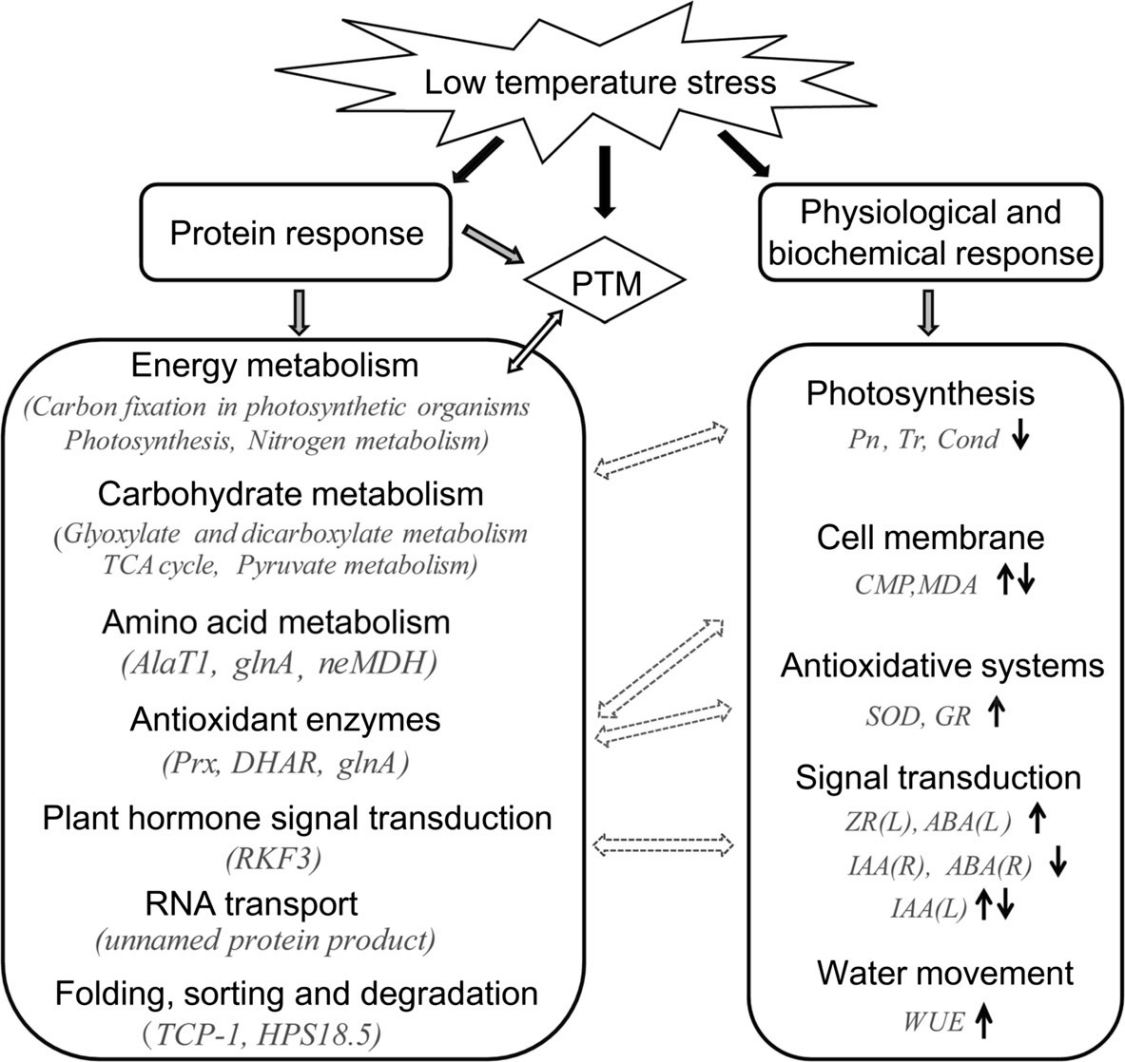
Overview of the physiological and biochemical responses, and proteome-wide responses. Details of the protein spots were described in Additional file 2: Tables S1 and S3. Red or green arrows indicated up-regulation or down-regulation under low-temperature stress, respectively

According to the findings, we proposed some scenarios involved in LT stress tolerance of *H. rhamnoides*. During the growth season, LT abiotic stress triggered signal transduction, stimulated genetic information processing (such as protein degradation and synthesis, and changes in enzymatic activity and hormone content), promoted cellular processes (cell damage, membrane lipid peroxidation and stomatal opening/closing) and regulated necessary metabolism pathways (especially the energy and carbohydrate metabolism mostly from photosynthesis). This then induced physiological (such as Pn, Tr, Cond, CMP and WUE) and biochemical changes (such as activity of SOD and GR, and contents of MDA and endogenous hormones), eventually leading to growth retardation of *H. rhamnoides* (Fig. 5). Additionally, post-translational modification (PTM), such as dephosphorylation (Fig. 3) might also play important roles in cold stress responses and adaptation in sea buckthorn [46].

## Conclusion

The responses of sea buckthorn to cold stress were complex and involved numerous physiological, molecular and cellular adaptations. The proteins involved in photosynthesis, plant hormone signal transduction, antioxidative systems and PTM played vital roles. Furthermore, the 2D-PAGE profile of sea buckthorn under LT stress provided useful candidate proteins for genetic improvement and understanding the general ability of plants to respond to a wide range of external environmental stresses.

## Methods

### Plant materials and treatments

Root turions of *H. rhamnoides* cv. ‘Chuisk’ were planted in 18 cm × 22 cm × 26 cm plastic pots containing 6 kg of a mixture of turfy soil/clay soil/sand (5:3:2). The plants were grown in a greenhouse under 25/20 °C of day/night temperatures, relative humidity of 60–70 % and CO_2_ concentration of 380 ± 10 μmol mol^−1^. After three months, the plants were divided into three groups/treatments (CK, T1 and T2), each with three biological replicates, applied as follows. CK was an unstressed control, T1 and T2 were 4 °C LT treatment, in which soil water content was maintained at about 30 % (i.e. 75–85 % of field moisture capacity) by regular watering during the whole experiment (i.e. from 0 to 14 d). Samples taken at 7 (T1) and 14 d (T2) were snap-frozen in liquid nitrogen, and stored at −80 °C for further studies. Soil water content was checked every 2 d using a moisture probe type HH2 Meter (Delta-T Devices Ltd., Cambridge, UK). For this, the plants were cultured separately in a 3.5 m × 2.2 m × 3.2 m AGC-2 Growth Cabinet (Zhejiang University Electric Equipment Factory, Hangzhou, China), whose parameters could be regulated strictly to be consistent with the greenhouse conditions.

### Measurement of physiological and biochemical parameters

Three fully expanded leaves in the middle portion of each stem were selected for measurement with a LI-6400 Portable Photosynthesis System (LI-COR Inc., Lincoln, NE, USA). Every measurement was repeated five times. The changes in Pn (μmol · m^−2^ · s^−1^), Tr (μmol · m^−2^ · s^−1^) and Cond (mol · m^−2^ · s^−1^) are shown in Fig. 1. The activities of major antioxidant enzymes (SOD and GR) (U · g^−1^), MDA (μmol · g^−1^) content were analyzed according to the method of Gao [47]. Molar conductances, used for CMP (%) were measured using a digital conductivity meter DDS-12DW Microprocessor (LIDA Instrument Factory, Shanghai, China). Fresh LA (cm^2^) was measured using a leaf area meter (CI-202, CID Inc., WA, USA). The content (ng · g^−1^) of four kinds of phytohormones (ABA, IAA, GA_3_ and ZR) were respectively measured in leaves and roots using enzyme-linked immunosorbent assay method. Each measure had three biological replicates.

### Protein extraction and quantification

Of frozen leaves, 2 g was homogenized in a pre-chilled mortar in 25 ml of cold acetone (−20 °C) containing 10 % (*V/V*) trichloroacetic acid and 65 mM dithiothreitol. The mixture was kept at −20 °C for 1 h, and then centrifuged at 12,000 *g* for 45 min. The supernatant was discarded and the pellet precipitated with 25 ml of cold acetone. After 1 h at −20 °C, the mixture was centrifuged at 12,000 *g* for 45 min, and the white pellet was lyophilized and stored at −80 °C. The protein pellet was dissolved in DIGE lysis buffer (7 M urea, 2 M thiourea, 4 % 3-[(3-chol-amidopropyl) dimethylammonio]-1-propane sulfonate and 0.2 % IPG buffer). The mixture was shaken and centrifuged at 12,000 *g* for 1 h. The supernatant was collected and protein concentrations were determined using the Bradford method with the protein assay reagent (Bio-Rad Laboratories, Hercules, CA, USA).

### Two-dimensional difference gel electrophoresis and imaging analysis

Nine protein samples (50 μg, pH 8.0–9.0) labeled with Cy3 and Cy5 dyes (labeled with Cy2 dye for use as internal standard for normalization) were mixed with a strip rehydration and then separated by 2D-polyacrylamide gel electrophoresis (PAGE). Total volume was adjusted to 50 μL, mixed 1:1 with DIGE lysis buffer and incubated for 30 min on ice in the dark. The reaction was then quenched with 10 mM lysine and additionally incubated for 10 min [48, 49]. Isoelectrofocusing was carried out with the IPGphor system (Amersham Pharmacia Biotech, Uppsala, Sweden) using Immobiline DryStrip gels (13 cm) with nonlinear pH gradients (pH 3–10). The IPGphor system was then programmed as follows: 12 h at 30 V, 1 h at 500 V, 1 h at 1000 V, 8 h at 8000 V and 4 h at 500 V. After IEF, the gel strips were incubated with equilibration buffer, and the strips were then transferred onto vertical 12.5 % (*w/v*) sodium dodecyl sulfate (SDS)-PAGE gels. The SDS-PAGE was performed on a Hoefer SE600 system (Amersham Pharmacia Biotech) with 15 mA/gel for 30 min and then 30 mA/gel until the bromophenol blue dye reached the bottom (about 0.5 cm) of the gels. The gels were scanned using an UMax Powerlook 2110XL laser scanner (Amersham Pharmacia Biotech), and the images analyzed using DeCyderTM 2D software (Amersham Pharmacia Biotech). Intra-gel spot detection, quantification and inter-gel matching and quantification were performed using Differential In-gel Analysis and Biological Variation Analysis modules respectively. To test for significant differences in expression of protein spots among pairwise groups, one-way analysis of variance (ANOVA) was performed at *p*-value ≤ 0.05. The differentially expressed protein spots were filtered based on an average volume ratio of twofold with significance at *p*-value ≤ 0.05. Each treatment had three biological replicates.

### In-gel trypsin digestion and MS analysis

These gels were fixed and stained with colloidal Coomassie brilliant blue (CBB). Proteins of interest, as defined by the DeCyder analysis TM 2D software, were excised from the CBB-stained gels for an in-gel trypsin digestion procedure [50]. The resolved peptides were analyzed on matrix-assisted laser desorption/ionization (MALDI) time-of-flight/time-of-flight (TOF/TOF) based on an MALDI TOF/TOF system (ultrafleXtreme, Bruker Daltonics, MA, USA). Protein identification was performed using the Mascot search engine version 2.1.03 (Matrix Science, London, UK) with a mass tolerance of 0.05 Da permitted for intact peptide masses and fragmented ions, and allowance for one missed cleavage in trypsin digests. Gln- > pyro-Glu (N-term Q), Oxidation (M) and Deamidated (NQ) as the potential variable modifications, Carbamidomethyl (C) as fixed modifications. The search that was performed using MASCOT v2.1.03 software (Matrix Science, London, UK) and the following settings: Uniprot_plant_database, one missed cleavage, fixed modifications of carbamidomethyl, variable modifications of oxidation, peptide tolerance 100 ppm, fragment mass tolerance 0.5 Da, peptide charge 1+. Only peptides with MS/MS ion scores significantly (*P* < 0.05) exceeding the MASCOT identity or extensive homology threshold were reported. Proteins were annotated using Gene Ontology (GO), Kyoto Encyclopedia of Genes and Genomes (KEGG) pathways. GO and pathway enrichment were analyzed using Gene Ontology Enrichment Analysis Software Toolkit (GOEAST) [20] and KEGG Orthology Based Annotation System (KOBAS) [21] Software Toolkit, respectively.

### Relative quantification of mRNA expression by RealTime PCR

Total RNAs from *H. rhamnoides* leaves in three groups were used for quantitative PCR analysis. Briefly, the first cDNA strands were obtained using a Thermo First cDNA Synthesis Kit, and were then subjected to quantification with 18S rRNA as an internal control using a standard SYBR Green PCR kit (Bio-Rad, Hercules, CA) on the StepOnePLUS RealTime PCR Detection System (Applied Biosystems, Foster City, CA). Quantitative PCR was then performed using the following conditions: 95 °C for 10 min; 40 cycles of 95 °C for 20 s and a 60 °C annealing temperature for 30 s; 95 °C for 15 s; 60 °C for 30 s; and 95 °C for 15 s. The primers for all 10 genes are listed in Online Additional file 2: Table S4. All reactions were performed in triplicate for each sample. Gene expression was quantified relative to 18S rRNA expression using the comparative cycle threshold (ΔCT) method. Differences in gene expression between the CK and LT stresses (T1 and T2) were detected by using the t-test.

## Additional files

Additional file 1: Figure S1. The 1D- and 2D- DIGE maps of H. rhamnoides leaves under control and low-temperature stress. (JPG 635 kb)
Additional file 2: Table S1A. The identified protein summary of H. rhamnoides cv. ‘Chuisk’ responding to low-temperature stress using MALDI-TOF-TOF. **Table S1B.** The identified protein detail of H. rhamnoides cv. ‘Chuisk’ responding to low-temperature stress using MALDI-TOF-TOF. **Table S2.** GO enrichment terms under low-temperature stress with Arabidopsis thaliana homologues using GOEAST. **Table S3.** KEGG pathways of protein spots under low-temperature stress. **Table S4.** Primers used for detecting the gene expression by reverse real-time PCR. (XLS 171 kb)
