# Supplementary figures and images for: Proteome profiling reveals insights into cold-tolerant growth in sea buckthorn
Source: Proteome Sci. 2016 Oct 7;14:14. doi: 10.1186/s12953-016-0103-z (PMC5054542; doi:10.1186/s12953-016-0103-z)

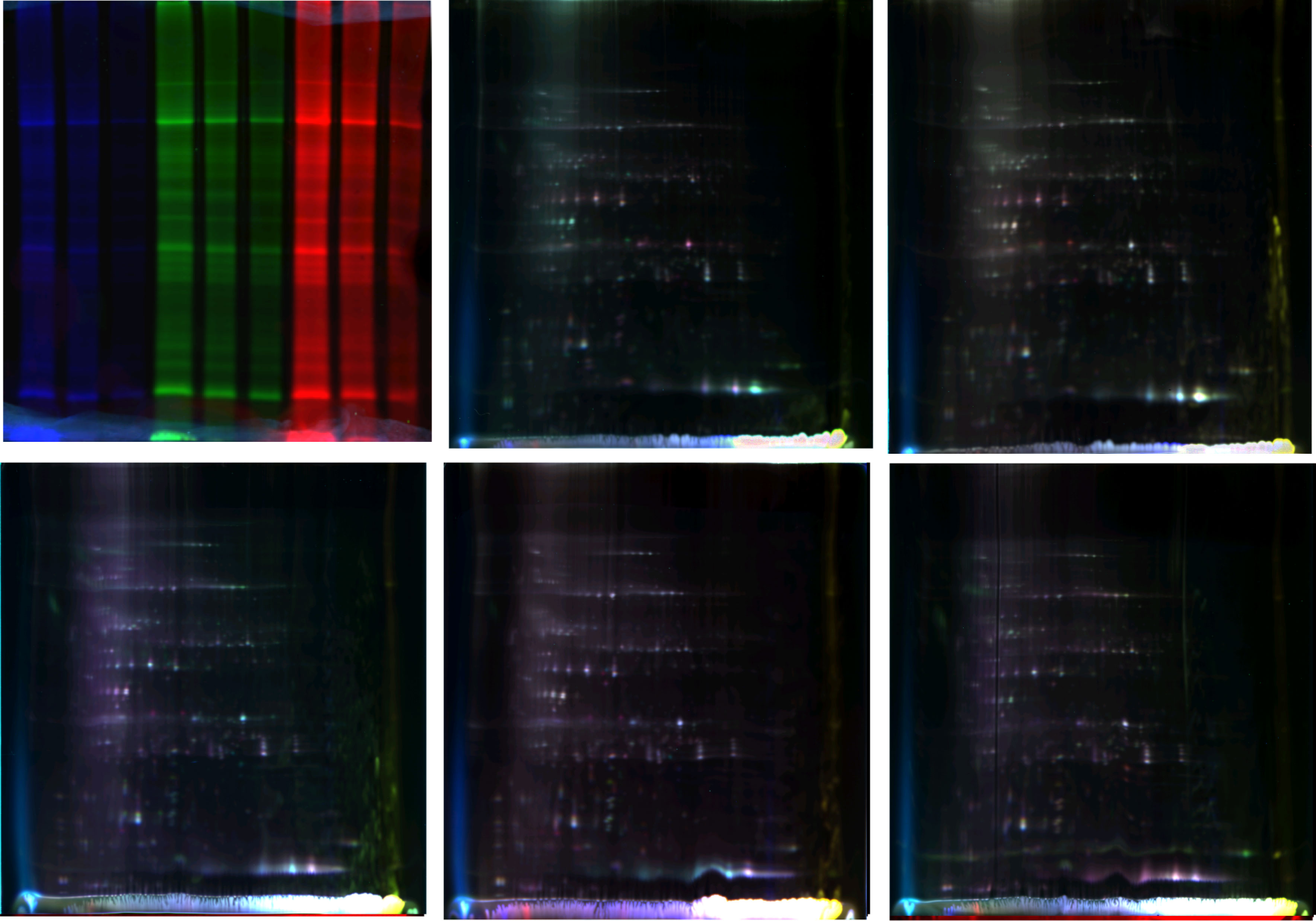

Supplement: Additional file 1: Figure S1. — The 1D- and 2D- DIGE maps of H. rhamnoides leaves under control and low-temperature stress. (JPG 635 kb) [file 12953_2016_103_MOESM1_ESM.jpg]
